# Estimated Cost-effectiveness of Subcutaneous Insulin Aspart in the Management of Mild Diabetic Ketoacidosis Among Children

**DOI:** 10.1001/jamanetworkopen.2022.30043

**Published:** 2022-09-06

**Authors:** Ibrahim Abdulaziz Bali, Muneera Rashid Al-Jelaify, Yazed AlRuthia, Jaazeel Zohair Mulla, Dana Fawzi Amlih, Alanoud Ibrahim Bin Omair, Reem Abdullah Al Khalifah

**Affiliations:** 1 Division of Pediatric Endocrinology, Department of Pediatrics, College of Medicine, King Saud University, Riyadh, Saudi Arabia; 2 Pharmacy Services, King Saud University Medical City, Riyadh, Saudi Arabia; 3 Department of Clinical Pharmacy, College of Pharmacy, King Saud University, Riyadh, Saudi Arabia; 4 Department of Pediatrics, College of Medicine, King Saud University, Riyadh, Saudi Arabia

## Abstract

**Importance:**

Intravenous (IV) insulin infusion is the standard of care for treating diabetic ketoacidosis (DKA) worldwide. Subcutaneous (SC) insulin aspart could decrease the use of health care resources.

**Objective:**

To compare the cost-effectiveness of mild uncomplicated DKA management with SC insulin aspart vs IV insulin infusion among pediatric patients from the perspective of a public health care payer using clinical data.

**Design, Setting, and Participants:**

This economic evaluation included children aged 2 to 14 years presenting to the emergency department of a single academic medical center with mild DKA between January 1, 2015, and March 15, 2020. The medical records for DKA treatment course and its associated hospitalization costs were reviewed. Data were analyzed from January 1, 2015, to March 15, 2020.

**Exposures:**

Subcutaneous insulin aspart vs IV regular insulin infusion.

**Main Outcomes and Measures:**

The incremental cost-effectiveness ratio (US dollars per hour), duration of DKA treatment, and length of hospital stay.

**Results:**

A total of 129 children with mild DKA episodes (mean [SD] age, 9.9 [3.1] years; 72 girls [55.8%]) were enrolled in the study. Seventy children received SC insulin aspart and 59 received IV regular insulin. Overall, the length of hospital stay in the SC insulin group was reduced (mean, 16.9 [95% CI, −31.0 to −2.9] hours) compared with the IV insulin group (*P* = .005). The mean (SD) cost of hospitalization in the SC insulin group (US $1071.99 [US $523.89]) was less than that in the IV insulin group (US $1648.90 [US $788.03]; *P* = .001). The incremental cost-effectiveness ratio was −34.08 (95% CI, −25.97 to −129.82) USD/h. The use of SC insulin aspart was associated with a lower likelihood of prolonged hospital stay (β = −17.22 [95% CI, −32.41 to −2.04]; *P* = .03) than IV regular insulin when controlling for age and sex.

**Conclusion and Relevance:**

Findings of this economic evaluation suggest that SC insulin aspart is dominant vs IV regular insulin in the management of mild uncomplicated DKA in children.

## Key Points

**Question:**

What is the cost-effectiveness of subcutaneous (SC) insulin aspart compared with standard-of-care intravenous insulin infusion in treating children with mild uncomplicated diabetic ketoacidosis (DKA)?

**Findings:**

This economic evaluation included 129 children with mild episodes of DKA. Subcutaneous insulin aspart was cost-effective in the treatment of children with mild DKA and was associated with faster DKA recovery, reduced length of hospital stay, and reduced overall costs of DKA treatment.

**Meaning:**

These findings suggest that SC aspart is a cost-effective strategy in the treatment of children with mild DKA.

## Teaser

This economic evaluation compares the cost-effectiveness associated with the use of subcutaneous insulin aspart vs intravenous insulin infusion for management of mild diabetic ketoacidosis in children.

## Introduction

Diabetic ketoacidosis (DKA) remains a leading cause of hospitalization among children with type 1 diabetes in Saudi Arabia and worldwide.^1,2,3,4,5^ Typically, its treatment requires intravenous (IV) regular insulin infusion and close clinical monitoring^6^ at a pediatric intensive care unit (ICU) or at a high-dependency unit setting, causing a burden on health care systems to accommodate other more acutely sick children.^7,8,9,10^ Use of health care resources is an important aspect of DKA management, especially in situations for which the standard-of-care management and monitoring facilities are not readily available either owing to limited health care resources or during epidemics.^6,7,10^

Subcutaneous (SC) rapid-acting insulin analogue has a similar efficacy and safety profile to IV regular insulin infusion for the treatment of children and adults with mild DKA^11,12,13,14,15,16^ who do not require ICU admission to receive care.^16^ Recently, the International Society for Pediatric and Adolescent Diabetes (ISPAD) suggested the use of SC rapid-acting insulin in the treatment of uncomplicated mild and moderate DKA in situations where continuous IV administration is not possible.^6,17^ However, the evidence of cost-effectiveness from the perspective of the public health care payer is limited.^12^ Thus, the practice of using SC insulin in treating mild uncomplicated DKA is still uncommon, even when ICUs worldwide experience shortages in bed availability, which limit access to health care.^16,18,19,20^

Given the high volume of DKA admissions at our institution and in accordance with the ISPAD recommendations, we adapted the practice of treating mild uncomplicated DKA with SC insulin since 2018.^6^ Therefore, this economic evaluation aims to compare the cost-effectiveness associated with using SC rapid-acting insulin vs IV regular insulin infusion for the management of mild DKA in children.

## Methods

### Ethical Considerations

We obtained approval for this economic evaluation from the King Saud University Institutional Review Board, which exempted the study from the need for informed consent owing to the use of retrospective data. The study procedures complied with the Good Clinical Practice and Declaration of Helsinki^21^ and followed the Consolidated Health Economic Evaluation Reporting Standards (CHEERS) reporting guideline.

### Population, Eligibility, and Data Collection

The study population comprised a retrospective cohort of patients treated at King Saud University Medical City. Our institution is a publicly funded, large, urban pediatric academic medical center with a 15-bed ICU capacity. We included children and adolescents (hereinafter referred to as children) with type 1 diabetes aged 2 to 14 years who visited the emergency department (ED) between January 1, 2015, and March 15, 2020, for mild DKA and received either SC insulin aspart or IV regular insulin infusion. We excluded children with complicated mild DKA, moderate DKA, severe DKA, hyperglycemia without DKA, or mild DKA treated with SC regular insulin, children visiting the ED for other causes, and children with type 2 diabetes.

Cases were identified using the electronic health system of the medical records by the *International Classification of Diseases, Ninth Revision* and *International Statistical Classification of Diseases and Related Health Problems, Tenth Revision* codes for ketoacidosis, type 1 diabetes, and hyperglycemia; in addition, all patients who received insulin treatment in the ED were identified. The diagnosis of mild DKA was established in the ED with a venous pH of 7.2 to 7.3, a bicarbonate level of 10 to 15 mEq/L (to convert to millimoles per liter, multiply by 1.0), and ketosis with either a blood β-hydroxybutyrate concentration of at least 31.2 mg/dL (to convert to millimoles per liter, multiply by 0.096) or urine ketones of at least 2 and a blood glucose level greater than 200 mg/dL (to convert to millimoles per liter, multiply by 0.0259).^6^

### Intervention and Comparison

Children who were treated with SC insulin aspart were compared with those treated with IV regular insulin infusion. Before January 2018, the standard of care for managing mild DKA in pediatric patients was IV regular insulin infusion with a dose of 0.1 U/kg/h. However, this treatment protocol was revised in January 2018 to be concordant with the ISPAD protocol, which recommends using SC insulin aspart injections for the treatment of mild uncomplicated DKA, whereas children presenting with impaired peripheral circulation were treated with IV regular insulin infusion.^6^ According to the ISPAD recommendations, after initial hydration in the first hour of management, the patient received an initial dose of 0.30 U/kg of insulin aspart, followed by subsequent doses of 0.15 to 0.20 U/kg every 2 hours until the resolution of DKA.^6^ The resolution of DKA is defined as a venous pH greater than 7.3 and bicarbonate level greater than 15 mEq/L.

During the study period, the rehydration fluids administration and monitoring of blood gas and electrolyte levels during a DKA episode were similar between both groups. Typically, vital signs and neurological vital signs are monitored hourly. Rehydration fluids containing 0.9% sodium chloride are administered as maintenance plus a 5% water deficit in addition to potassium replacement according to the potassium level and the state of renal function.^6^ Glucose level is monitored hourly at bedside using a glucometer, and venous blood gas and serum electrolyte levels are monitored every 2 to 4 hours. If the serum glucose level is less than 300 mg/dL, 5% dextrose is added to the IV fluid.^6^ In our institution according to bed availability, children with DKA are often treated in the ED or the ICU owing to the limited availability of 1:1 nursing care in the pediatric ward.

After the resolution of DKA, the patient is transitioned to the usual daily regimen of SC insulin, or, if newly diagnosed, the regimen will be calculated based on their weight and age. The patient then will be either transferred to the ward or discharged based on the need for further monitoring, education, or treatment of an infection as an inpatient.

### Measures

We reviewed the medical records of included patients for baseline demographic characteristics and DKA treatment course, including the duration of management (calculated from the time the patient was seen by the ED triage nurse until management of DKA was stopped). The time when DKA management was stopped was defined as when results of the first venous blood gas measurement confirmed that the patient was recovered from DKA. We calculated the time to bolus from the time the patient was seen by the ED triage nurse until the IV fluid bolus was given and the time to insulin from the time the patient was seen by the ED triage nurse until insulin management was started. Furthermore, we evaluated the blood glucose level trend during DKA management using the blood gas and electrolyte levels at initial presentation and on resolution of DKA. The duration of admission was calculated from the time the patient was seen by the ED triage nurse until the time of discharge from the hospital, which was identified using the nursing discharge note.

We calculated the hospitalization cost based on the cost of health care resources, including the cost of hospital stay (ED, ICU, and ward), glucose strips, laboratory reagents, IV fluid, insulin, and various medications (eg, antiemetics, antibiotics, or laxatives) that were used during admission. All costs are based on the Saudi Ministry of Health standard costs. We did not include the costs of personnel, laundry, nutrition, nursing, housekeeping services, or the parents’ absence from work. There are no direct costs paid by the patients and their families.

### Outcomes

The primary outcome consisted of the cost-effectiveness of SC insulin vs IV regular insulin infusion in the management of mild DKA.^22,23^ The secondary outcomes included DKA management outcomes, which included the length of hospital stay (LOS), the duration of DKA management, and DKA management complications, such as acute kidney injury (AKI). We defined AKI as a serum creatinine value of more than 1.5 times the estimated baseline serum creatinine level according to the Kidney Disease: Improving Global Outcomes group.^24^ We estimated the baseline serum creatinine value using the Schwartz estimating equation with an estimated glomerular filtration rate of 120 mL/min/1.73m^2^. Furthermore, we looked for factors that might increase the LOS after DKA resolution.

### Statistical Analysis

Data were analyzed from January 1, 2015, to March 15, 2020. The study sample size of 116 was estimated based on the expected reduction of the hospitalization cost by 10%, assuming an α of .05 and a power of 80% for a 2-sided test. Previous data indicated that the mean cost of DKA treatment is US $1835.75 (95% CI, $1482.36 to $2187.81).^25^

We presented the continuous data as mean with SD or as median with IQR depending on the distribution of the data, whereas categorical data were presented as frequencies and proportions. We calculated the incremental cost-effectiveness ratio as the difference in total hospitalization cost (in USD) divided by the difference in the LOS between the SC and IV insulin groups (in hours). We used the unpaired 2-tailed *t* test to compare the differences between the means of the 2 groups and Pearson χ^2^ test to assess the difference in categorical data. We performed multiple linear regression to examine the association between the LOS in hours and the use of IV regular insulin vs SC insulin aspart in the management of DKA when controlling for age and sex. To examine the cost-effectiveness of SC vs IV insulin, we conducted an inverse probability weighting with a propensity score based on age and sex and bootstrapping with 10 000 replications to generate the 95% CIs for both the outcome (eg, LOS in hours) and cost. Statistical analyses were conducted using SAS, version 9.4 (SAS Institute Inc), and STATA/SE, version 16.1 for Mac (StataCorp LLC). Two-sided *P* < .05 indicated statistical significance.

## Results

**Figure 1.**
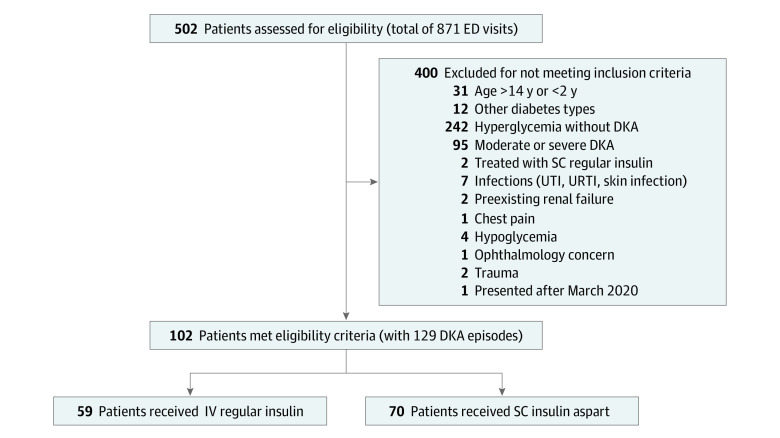
Study Flow Diagram DKA indicates diabetic ketoacidosis; ED, emergency department; IV, intravenous; SC, subcutaneous; URTI, upper respiratory tract infection; and UTI, urinary tract infection.

A total of 502 children with diabetes (871 visits) visited the ED during the study period. A total of 129 mild DKA episodes occurred among children with a mean (SD) age of 9.9 (3.1) years who met the inclusion criteria (57 boys [44.2%] and 72 girls [55.8%]). We excluded 400 children (742 visits), of whom 95 had moderate or severe DKA and 242 had hyperglycemia without ketoacidosis; 63 were excluded for other reasons (Figure 1). Among the 129 mild DKA episodes, 70 were treated with SC insulin aspart and 59 were treated with IV regular insulin. Of those, 21 children in the SC insulin group were treated before 2018 to assess staff comfort level and establish local experience, and 4 received IV regular insulin during the first few months of implementing the SC insulin treatment protocol. The analysis was based on the allocated treatment.

The mean duration of diabetes was 4.1 (2.9) years, and 23 patients (17.8%) had newly diagnosed diabetes. The number of mild DKA episodes for each patient, baseline biochemical data on admission, and number of patients with AKI were similar in both treatment groups. Insulin omission was the most frequent cause of DKA (39 [30.2%]), followed by infection (30 [23.3%]). In 37 DKA episodes (28.7%), the cause was not documented in the medical records (Table 1). The use of SC insulin aspart was associated with a lower likelihood of prolonged hospital stay (β = −17.22 [95% CI, −32.41 to −2.04]; *P* = .03) than that of IV regular insulin infusion when controlling for age and sex (eTable 1 in the Supplement). There was no significant difference across both groups for causes leading to prolonged LOS after DKA resolution (eTable 2 in the Supplement).

**Table 1.** Baseline Characteristics

Variable	Study treatment^a^			Mean difference (SEM)	*P* value
	All (n = 129)	IV group (n = 59)	SC group (n = 70)		
Age, years	9.9 (3.1)	9.9 (2.9)	10.1 (3.2)	0.2 (0.5)	.69
Sex, No. (%)					
Boys	57 (44.2)	25 (43.4)	32 (45.7)	NA	.70
Girls	72 (55.8)	34 (57.6)	38 (54.3)	NA	
Weight, kg	31.5 (12.1)	32.1 (12.4)	30.9 (11.9)	1.3 (2.1)	.55
Cause of DKA, No. (%)					
Insulin omission	39 (30.2)	16 (27.1)	23 (32.9)	NA	.48
Infection	30 (23.3)	8 (13.6)	22 (31.4)	NA	.02
Newly diagnosed	23 (17.8)	12 (20.3)	11 (15.7)	NA	.49
Not documented	37 (28.7)	24 (40.7)	13 (18.6)	NA	.006
Other^b^	5 (3.9)	1 (1.7)	4 (5.7)	NA	.24
Diabetes duration, y	4.1 (2.9)	4.5 (3.2)	3.8 (2.8)	0.7 (0.7)	.37
No. of DKA episodes for each child	1.3 (0.7)	1.3 (0.8)	1.3 (0.6)	0.7 (0.1)	.59
Time to bolus, h	1.5 (1.1)	1.6 (1.1)	1.5 (1.3)	0.1 (0.2)	.78
Time to insulin administration, h	3.9 (1.8)	3.9 (1.9)	3.8 (1.7)	0.1 (0.3)	.67
pH	7.2 (0.3)	7.2 (0.03)	7.25 (0.04)	0.01 (0.0)	.45
Pco_2_, mm Hg	28.2 (6.0)	27.6 (5.9)	28.6 (6.2)	1.0 (1.1)	.38
Serum bicarbonate level, mEq/L	13.9 (1.8)	13.4 (1.5)	14.4 (1.9)	1.0 (0.3)	.001
Serum creatinine level, mg/dL	0.7 (0.2)	0.8 (0.2)	0.7 (0.2)	0.04 (0.03)	.21
Serum urea nitrogen level, mg/dL	18.2 (25.8)	21.6 (37.5)	15.1 (4.5)	6.4 (4.5)	.16
Serum sodium level, mEq/L	131.9 (3.8)	131.4 (4.0)	132.3 (3.6)	0.9 (0.7)	.16
Serum potassium level, mEq/L	4.5 (0.6)	4.5 (0.7)	4.5 (0.6)	−0.1 (0.1)	.47
Serum chloride level, mEq/L	94.7 (8.0)	94.8 (4.3)	94.5 (10.2)	0.3 (1.4)	.82
Serum glucose level, mg/dL	381.6 (122.4)	399.6 (124.2)	367.2 (120.6)	32.4 (72.0)	.14
Serum phosphorus level, mg/dL	4.6 (1.2)	4.6 (1.2)	4.6 (1.2)	0.1 (0.3)	.83
Serum magnesium level, mg/dL	1,9 (0.2)	1.9 (0.5)	1.9 (0.2)	0.1 (0.2)	.72
Serum osmolality, mOsm/kg	290.0 (9.9)	289.9 (11.4)	291.7 (8.6)	1.8 (1.8)	.33
AKI at baseline, No. (%)^c^	76 (58.9)	35 (59.3)	41 (58.6)	NA	.48

Abbreviations: AKI, acute kidney injury; DKA, diabetic ketoacidosis; IV, intravenous; NA, not applicable; SC, subcutaneous.

SI conversion factors: To convert bicarbonate to mmol/L, multiply by 1; chloride to mmol/L, multiply by 1; creatinine to μmol/L, multiply by 88.4; glucose to mmol/L, divide by 18; magnesium to mmol/L, multiply by 0.4114; phosphorus to mmol/L, multiply by 0.323; potassium to mmol/L, multiply by 1; serum osmolality to mmol/kg, multiply by 1; sodium to mmol/L, multiply by 1; and urea nitrogen to mmol/L, multiply by 0.357.

^a^ Unless otherwise indicated, data are expressed as mean (SD).

^b^ Includes inappropriate insulin storage, wrong technique of insulin injection, and improper dosage with fasting of Ramadan.

^c^ Six children had a missing AKI outcome because of missing height data.

**Figure 2.**
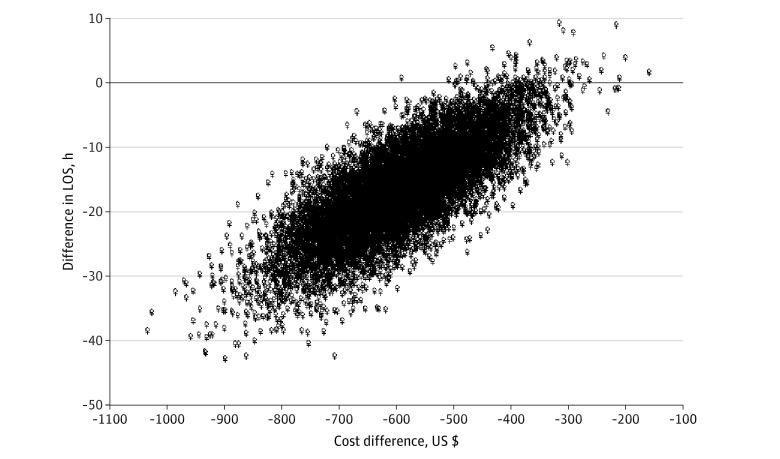
Probabilistic Sensitivity Analysis of the Cost-effectiveness of Subcutaneous Insulin Aspart vs Intravenous Regular Insulin Infusion in the Management of Diabetic Ketoacidosis LOS indicates length of hospital stay.

The mean (SD) cost of managing DKA using SC insulin aspart was US $1071.99 (US $523.89) in contrast to US $1648.90 (US $788.03) (*P* = .001) for IV regular insulin. The cost breakdown for each treatment group is shown in eFigure 1 in the Supplement. The use of SC insulin aspart was associated with a mean lower cost (US −$577.90 [95% CI, −$804.48 to −$379.96]) and shorter hospital stay (16.9 [95% CI, −31.0 to −2.9] hours; *P* = .005) (Table 2 and Table 3), with an incremental cost-effectiveness ratio of −34.08 (95% CI, −25.97 to −129.82) USD/h than that of IV regular insulin infusion (Figure 2). A total of 21 patients were admitted to the ICU, among whom 18 were from the IV regular insulin group, whereas the rest of the IV group was treated in the ED either owing to a lack of available ICU beds or if the DKA resolved before an ICU bed was available. Three patients from the SC insulin group were admitted to the ICU. The odds ratio of ICU admission was 0.11 (95% CI, 0.02-0.39). Treatment for 2 patients from the SC group was switched to IV regular insulin infusion owing to worsening of blood gas levels, and these patients were moved to the ICU, whereas 1 patient was admitted to the ICU for observation during the first month of starting the SC insulin protocol for mild DKA.

**Table 2.** Mean Length of Hospital Stay and Costs of SC Insulin Aspart and IV Regular Insulin Infusion for Management of Mild Diabetic Ketoacidosis

Outcome	Treatment group		Mean difference (95% CI)
	SC insulin aspart	IV regular insulin infusion	
Cost of treatment, mean (SD), US $	1071.99 (523.89)	1648.90 (788.03)	−577.9 (−804.48 to −379.96)
Length of hospital stay, mean (SD), h	45.79 (37.26)	62.72 (50.00)	−16.93 (−30.98 to −2.93)
Incremental cost-effectiveness ratio (95% CI), USD/h	−34.08 (−25.97 to −129.82)	NA	NA

Abbreviations: IV, intravenous; NA, not applicable; SC, subcutaneous.

**Table 3.** Treatment Outcomes of Mild DKA

Outcome	Study treatment^a^			Mean difference (SEM)	*P* value
	All (n = 129)	IV group (n = 59)	SC group (n = 70)		
Duration of DKA treatment, h	10.3 (5.8)	11.9 (7.0)	9.1 (3.9)	2.8 (1.0)	.005
Length of hospital stay, h	53.5 (44.2)	62.7 (50.0)	45.8 (37.3)	16.9 (7.7)	.03
New-onset T1D length of hospital stay, h	91.6 (32.3)	87.9 (20.5)	95.7 (42.4)	7.8 (13.7)	.57
Children requiring ICU admission, No. (%)	21 (16)	18 (85.7)	3 (14.3)	NA	.001
Length of ED stay, h	14.1 (7.8)	14.3 (7.9)	14.0 (7.7)	0.2 (1.4)	.89
Length of ICU stay, h	20.5 (14.5)	22.1 (14.9)	11.0 (7.6)	11.1 (8.9)	.23
Length of ward stay, h	33.1 (42)	39.0 (47.0)	28.2 (36.9)	10.9 (7.3)	.14
Cost of ED stay, US $	725.94 (597.14)	729.10 (597.60)	723.20 (601.10)	−5.88 (105.90)	.95
Cost of ICU stay, US $	209.11 (596.33)	422.10 (817.60)	29.55 (162.20)	−392.60 (99.90)	<.001
Cost of ward stay, US $	856.20 (1085.49)	1008.40 (1214.20)	727.90 (954.00)	−280.50 (191.00)	.14
Total cost of IV fluids and insulin, US $	107.05 (42.54)	135.40 (38.27)	83.17 (29.36)	−52.20 (6.00)	<.001
Cost of medications, US $^b^	10.17 (10.70)	9.53 (12.07)	10.79 (9.39)	−1.25 (1.90)	.67
Cost of investigations, US $	182.49 (105.01)	217.80 (106.40)	152.70 (94.71)	−65.11 (17.70)	<.001
No. of times of glucose level measurement, mean (SD)	9.9 (3.7)	9.9 (3.6)	9.9 (3.9)	0.02 (0.7)	.97
pH	7.3 (0.3)	7.3 (0.3)	7.3 (0.3)	−0.003 (0.0)	.67
Pco_2_, mm Hg	32.0 (5.3)	31.0 (5.6)	32.9 (4.9)	−1.8 (0.9)	.05
Serum bicarbonate level, mEq/L	18.1 (1.9)	17.8 (1.8)	18.4 (1.9)	−0.6 (0.3)	.07
Serum creatinine level, mg/dL	0.6 (0.2)	0.6 (0.2)	0.6 (0.2)	0.1 (0.03)	.06
Serum urea nitrogen level, mg/dL	12.0 (4.5)	12.0 (4.5)	11.8 (4.8)	0.3 (0.8)	.70
Serum sodium level, mEq/L	136.6 (3.5)	136.7 (3.4)	136.5 (3.5)	0.2 (0.6)	.77
Serum potassium level, mEq/L	4.1 (0.5)	4 (0.6)	4.2 (0.4)	0.1 (0.1)	.15
Serum chloride level, mEq/L	104 (4.3)	104.4 (3.9)	103.7 (4.8)	0.7 (0.8)	.42
Serum glucose level, mg/dL	219.6 (93.6)	203.4 (90.0)	235.8 (93.6)	32.4 (18.0)	.07
Serum phosphorus level, mg/dL	3.7 (0.9)	3.4 (0.9)	4.0 (0.9)	0.5 (0.3)	.07
Serum magnesium level, mg/dL	1.7 (0.5)	1.7 (0.5)	1.7 (0.2)	0 (0.2)	.93
Serum osmolality, mOsm/kg	285.9 (26.2)	282.2 (34.9)	289.9 (10.5)	7.7 (4.9)	.12
Unresolved AKI, No. (%)^c^	29 (22.5)	17 (28.8)	12 (17.1)	NA	.06
Hypoglycemia, No. (%)	18 (13.9)	7 (11.9)	11 (15.7)	NA	.50
Lowest glucose level, mg/dL	52.2 (12.6)	61.2 (7.6)	46.8 (12.6)	14.4 (5.4)	.03
Hypokalemia, No. (%)	17 (0.8)	10 (16.9)	7 (10.0)	NA	.25
Lowest potassium level, mEq/L	3.2 (0.2)	3.1 (0.2)	3.2 (0.2)	0.9 (0.1)	.38

Abbreviations: AKI, acute kidney injury; DKA, diabetic ketoacidosis; ED, emergency department; ICU, intensive care unit; IV, intravenous; NA, not applicable; SC, subcutaneous; T1D, type 1 diabetes.

SI conversion factors: To convert bicarbonate to mmol/L, multiply by 1; chloride to mmol/L, multiply by 1; creatinine to μmol/L, multiply by 88.4; glucose to mmol/L, divide by 18; magnesium to mmol/L, multiply by 0.4114; phosphorus to mmol/L, multiply by 0.323; potassium to mmol/L, multiply by 1; serum osmolality to mmol/kg, multiply by 1; sodium to mmol/L, multiply by 1; and urea nitrogen to mmol/L, multiply by 0.357.

^a^ Unless otherwise indicated, data are expressed as mean (SD).

^b^ Includes medications such as antibiotics, antiemetics, laxatives, etc.

^c^ Six children were missing an AKI outcome because of missing height data.

### DKA Management Outcome and Complications

The treatment of mild DKA with SC insulin aspart was associated with a shorter duration of DKA management than use of IV regular insulin by a mean (SD) of 2.83 (11.24) hours despite similarities in the time from presentation to the ED until starting fluid bolus and insulin. The biochemical profile at DKA resolution was similar between both groups (Table 3). The frequency of glucose level measurement, rate of glucose level decline after starting DKA management, and hypoglycemic episodes were all similar between both groups. eFigure 2 in the Supplement shows the mean hourly change in glucose levels during treatment between the groups.

Mild adverse effects of treatment in the form of hypoglycemia and hypokalemia were seen in 18 patients (13.9%). The SC group had fewer patients with AKI compared with the IV group on DKA resolution, but the difference was not statistically significant (12 [17.1%] vs 17 [28.8%]; *P* = .06). No deaths or cerebral edema was reported in any of the patients in both treatment groups.

## Discussion

The use of SC insulin aspart in the treatment of children with mild DKA at our center was associated with reduction of hospitalization costs and improved efficacy of DKA management compared with use of IV regular insulin, with savings of US $34.08 per hour from the Saudi public health care payer’s perspective. Children who received SC insulin had their DKA resolved 2.83 hours earlier and had a shorter LOS by 16.9 hours. The reduced overall cost of hospitalization in the SC group may be associated with the reduced need for ICU admission, shorter LOS, and reduced cost of IV fluids, insulin, and diagnostics. Similarly, Umpierrez et al^12^ reported a 39% reduction in hospitalization cost related mainly to ICU admission; however, they did not report the incremental cost-effectiveness ratio nor the actual included costs in the cost estimate.

As reported in previous randomized clinical trials comparing the 2 treatment modalities for children and adults with uncomplicated mild DKA,^12,13,14,15,26^ SC insulin is safe and effective. The mean LOS in the SC insulin group was 1.90 days, and the mean duration of DKA treatment was 9.06 hours. In comparison, in the IV regular insulin group, the reported mean DKA treatment duration was 10.38 to 13.00 hours, and the mean LOS was 3.33 days.^14^ The observed differences between our study and previous reports could be attributed to the variability in SC insulin dosing regimens used in each study in comparison with the unified standard dose of IV regular insulin infusion. In addition, our study included a larger sample size, which improved the precision of the effect estimate. The frequency of adverse effects was similar to the frequency observed in other studies.^12,13,14,15,16^

Despite the observable reduction in DKA treatment duration and LOS in the SC insulin group, children remain admitted for a significant time. The causes leading to prolonged admission after DKA resolution were similar between groups. Similarly, previous studies have reported prolonged admission after DKA resolution.^12,13,14,27,28,29^ Future interventions are needed to reduce prolonged hospitalization after DKA resolution.

Although SC insulin does not require preparation and is easier to administer, the recurring injections every 2 hours can be painful and frustrating to the child and add to the discomfort caused by laboratory monitoring every 2 to 4 hours. The use of a flexible SC catheter can help to overcome this disadvantage of using SC insulin injection without affecting its efficacy.^30^ Moreover, the recent ISPAD guideline for management of DKA during COVID-19 raised concern about a possible increased burden on the nursing staff due to the frequent injections.^17^ In our study, the SC insulin group had their DKA resolved 2.83 hours sooner than the IV group, therefore saving nursing effort in extracting blood for laboratory monitoring and continuing DKA monitoring and management.^11,31,32^

The actual duration of DKA treatment may be reduced further if insulin aspart is readily available in the ED medication stock. Typically, it took approximately 2.5 hours for insulin administration to be started after IV fluid administration in both groups because the pharmacy has to prepare the insulin before administration. When it comes to pharmacy-related obstacles, the use of IV regular insulin infusion has many drawbacks compared with SC insulin. Insulin is a high-alert medication and is susceptible to human error.^32^ Human error has been traced back to knowledge lapses among different pharmacy staff level owing to the different insulin concentrations and products, differences between insulin syringes and other parenteral syringes, and a perceived urgency with treating DKA.^31^ Moreover, pharmacists have a heavy workload, including the verification, preparation, and labeling of orders. Pharmacists working at the evening and night shifts experience most of these problems owing to limitations in human resources.^11,31^

### Limitations

Our study has some limitations. First, this was an economic evaluation using a retrospective cohort; we did not collect data on health-related quality of life (HRQOL). Although DKA clearly leads to lower HRQOL among children, there is no evidence linking ICU admission with HRQOL.^33^ It is reasonable to assume that reduced rates of ICU admission and faster DKA resolution with SC insulin aspart would result in a positive HRQOL. A growing body of literature has focused on the persistent psychologic distress caused by ICU admission on parents and children.^34,35,36^ Future research is needed to evaluate the societal perspective on interventions that affect HRQOL during DKA treatment and to evaluate strategies to minimize the burden of DKA. Second, our cost analyses included costs before and after DKA resolution, which might have inflated the cost of DKA management. Third, our study reflects the perspective of a single health care system with a small number of patients, although the DKA treatment costs and LOS at our center were similar to those of most public health care institutions in Saudi Arabia. The median LOS for mild DKA in Saudi Arabia is 2 to 4 days, whereas in the US health care system it is 3.22 days.^27,28,29^ Moreover, the costs of different health care resources used in the management of DKA are not significantly different from those of other health care systems with universal health care, such as the UK public health care system.^25^ Future studies are needed to assess the generalizability of our findings to other market-based health care systems, such as the US health care system, in which DKA treatment is costly.^10^

## Conclusions

The clinical data of this economic analysis provide evidence from the perspective of a public health care payer suggestive of the feasibility and cost-effectiveness of using SC insulin aspart in the treatment of children with uncomplicated mild DKA in a tertiary setting with good health care resources. Pediatricians, endocrinologists, emergentologists, intensivists, and policy makers may need to reconsider the usual practice of using IV regular insulin for mild DKA.

## Notes

Supplement. **eTable 1.** Multiple Linear Regression of the Association Between the Use of SC Aspart vs IV Regular Insulin and the Length of Hospital Stay **eTable 2.** Causes of Prolonged Length of Hospital Stay **eFigure 1.** The Cost Breakdown per Patient Treated With IV Insulin Infusion and SC Aspart **eFigure 2.** The Mean Hourly Glucose Changes During DKA Treatment
