# Supplementary material for: Estimated Cost-effectiveness of Subcutaneous Insulin Aspart in the Management of Mild Diabetic Ketoacidosis Among Children
Source: JAMA Netw Open. 2022 Sep 6;5(9):e2230043. doi: 10.1001/jamanetworkopen.2022.30043 (PMC9449786; doi:10.1001/jamanetworkopen.2022.30043)
Supplement: Supplement. — eTable 1. Multiple Linear Regression of the Association Between the Use of SC Aspart vs IV Regular Insulin and the Length of Hospital Stay eTable 2. Causes of Prolonged Length of Hospital Stay eFigure 1. The Cost Breakdown per Patient Treated With IV Insulin Infusion and SC Aspart eFigure 2. The Mean Hourly Glucose Changes During DKA Treatment [file jamanetwopen-e2230043-s001.pdf]

Supplementary Online Content

Bali IA, Al-Jelaify MR, AlRuthia Y, et al. Estimated cost-effectiveness of subcutaneous insulin aspart in the management of mild diabetic ketoacidosis among children. *JAMA Netw Open*. 2022;5(9):e2230043. doi:10.1001/jamanetworkopen.2022.30043

eTable 1. Multiple Linear Regression of the Association Between the Use of SC Aspart vs IV Regular Insulin and the Length of Hospital Stay

eTable 2. Causes of Prolonged Length of Hospital Stay

eFigure 1. The Cost Breakdown per Patient Treated With IV Insulin Infusion and SC Aspart

eFigure 2. The Mean Hourly Glucose Changes During DKA Treatment

This supplementary material has been provided by the authors to give readers additional information about their work.

©2022 Bali IA et al. *JAMA Network Open*.

eTable 1. Multiple Linear Regression of the Association Between the Use of SC Aspart vs IV Regular Insulin and the Length of Hospital Stay

**Variable**	**β-estimate**	***p*-value**	**95% Confidence Limits**	
			**Lower limit**	**Upper limit**
SC insulin Aspart	-17.22	0.0265	-32.41	-2.044
Age	- 0.72	0.569	-3.228	1.782
Female sex	-13.55	0.084	-28.96	1.852

©2022 Bali IA et al. *JAMA Network Open*.

eTable 2. Causes of Prolonged Length of Hospital Stay					
	Value, No.(%)				
**Variables**	**Overall (n=129)**	**IV (n=59)**	**S.C (n=70)**	**Mean difference**	**P-value**
**Education**	54 (41.8)	26 (44.1)	28 (40)	NA	.64
**Treatment of infection**	15 (11.6)	3 (5.1)	12 (17.1)	NA	.033
**Social reasons**	4 (0.3)	2 (3.3)	2 (2.9)	NA	.86
**Weekend admission**	4 (0.3)	2 (3.3)	2 (2.9)	NA	.86
**insulin dose adjustment**	3 (0.2)	0(0)	3 (4.3)	NA	.1
**Other causes a**	8 (0.6)	5 (8.5)	3(4.3)	NA	.33
**No. of diabetic education sessions in each admission, mean (SD)**	1.6 (1.6)	1.9 (1.6)	1.4 (1.6)	0.4	.12
**No. of dietitian education sessions in each admission, mean (SD)**	0.5 (0.6)	0.6 (0.7)	0.5 (0.6)	0.1	.32

a other causes: gastroenteritis, electrolyte imbalance, follow up of laboratory results

©2022 Bali IA et al. *JAMA Network Open*.

eFigure 1. The Cost Breakdown per Patient Treated With IV Insulin Infusion and SC Aspart

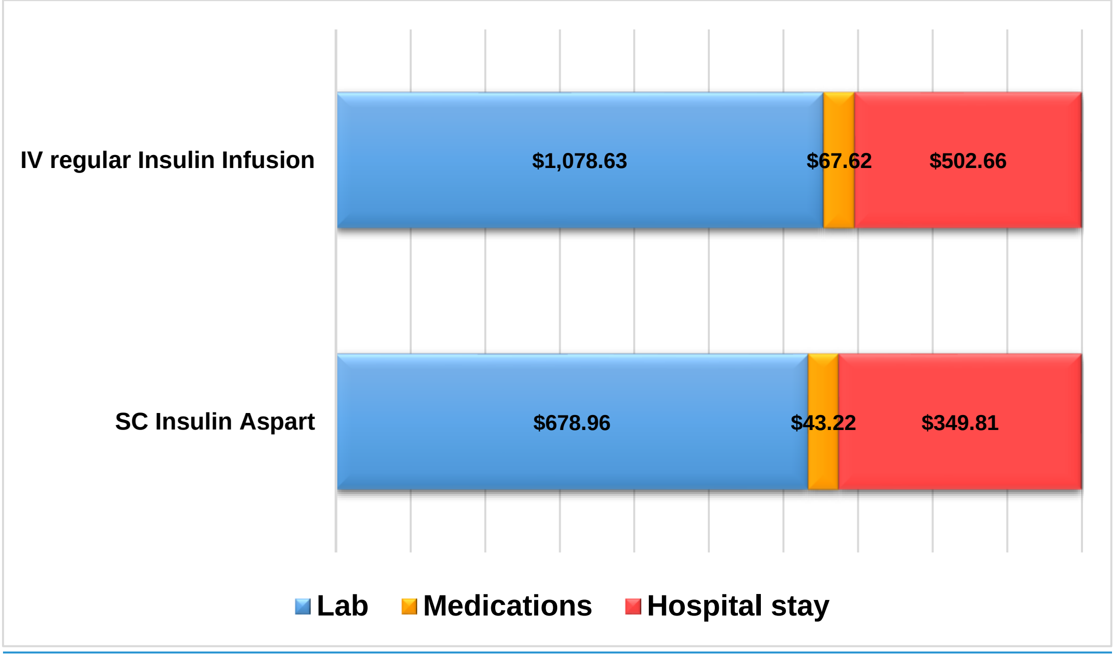

eFigure 1. The Cost Breakdown per Patient Treated With IV Insulin Infusion and SC Aspart

©2022 Bali IA et al. *JAMA Network Open*.

eFigure 2. The Mean Hourly Glucose Changes During DKA Treatment

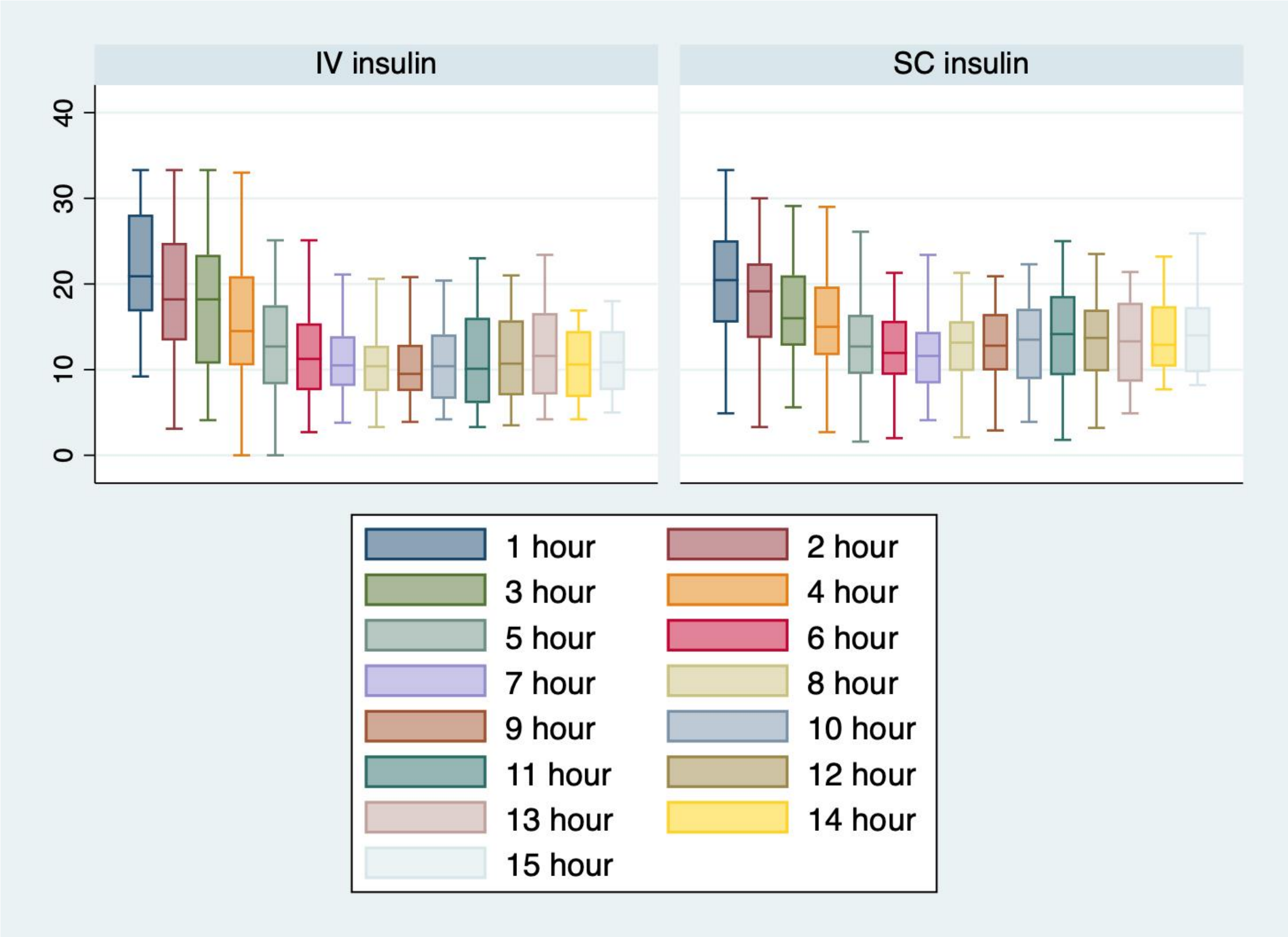

©2022 Bali IA et al. *JAMA Network Open*.
